# Expression of the *HOXA* gene family and its relationship to prognosis and immune infiltrates in cervical cancer

**DOI:** 10.1002/jcla.24015

**Published:** 2021-10-04

**Authors:** Fenfen Ge, Weiwei Tie, Junli Zhang, Yingying Zhu, Yingying Fan

**Affiliations:** ^1^ Department of Gynecology Ningbo Medical Center Lihuili Hospital Ningbo University Ningbo China

**Keywords:** cervical cancer, diagnosis, *HOXA* family, ImmuCellAI, immunity biomarkers, prognosis

## Abstract

**Background:**

The homeobox A cluster (*HOXA*) gene family is participated in multiple biological functions in human cancers. To date, little is known about the expression profile and clinical significance of *HOXA* genes in cervical cancer.

**Methods:**

We downloaded RNASeq data of cervical cancer from The Cancer Genome Atlas (TCGA) database. The difference in *HOXA* family expression was analyzed using independent samples *t* test. Cox proportional hazard regression analysis was used to assess the effect of *HOXA* family expression on survival, and a nomogram predicting survival was generated. We assessed the infiltration difference in immune cells and expression difference of immunity biomarkers between two groups with different expression level of *HOXA* genes through Immune Cell Abundance Identifier (ImmuCellAI) and independent samples *t* test, respectively.

**Results:**

Our results showed that the *HOXA1* gene was upregulated, while the *HOXA10* and *HOXA11* were downregulated in cervical cancer. Downregulation of *HOXA1* was related to a poor outcome for cervical cancer patient. We also identified a significantly increased abundance of T helper 2 cells (Th2) and higher expression of PD‐L1 in cervical cancer patients with lower expression of *HOXA10* and *HOXA11*. The gene set enrichment analysis (GSEA) results indicated that *HOXA1* and *HOXA11* were involved in immune responses pathways and participated in the activation of a variety of classic signaling pathways related to the progression of human cancer.

**Conclusion:**

This study comprehensively analyzed different *HOXA* genes applying public database to determine their expression patterns, potential diagnostic, prognostic, and treatment values in cervical cancer.

## Graphical abstract

Our study comprehensively analyzed different *HOXA* genes applying public database to determine their expression patterns, potential diagnostic, prognostic, and treatment values in cervical cancer. Firstly, we compared the expression of *HOXA* members in cervical cancer than normal controls, and results showed only three *HOXA* members with statistical significance (*HOXA1*, *HOXA10,* and *HOXA11*). Then, the prognostic potential was assessed by the following steps: (1) Kaplan‐Meier plot showed low expression of seven *HOXA* members was correlated with favor overall survival of cervical cancer (including *HOXA1*, *HOXA2*, *HOAX3*, *HOXA4*, *HOXA5*, *HOXA6,* and *HOXA9*). (2) Univariate Cox proportional hazards regression analysis confirmed the prognostic value of *HOXA1*, *HOXA2*, *HOXA3*, and *HOXA4*. (3) Multivariate Cox proportional hazards regression analysis determined the prognostic value of *HOXA1* and *HOXA3*. Eventually, the *HOXA1* could be served as an independent prognostic biomarker for poor outcome of cervical cancer. Subsequently, a significantly increased abundance of T helper 2 cells (Th2) and higher expression of PD‐L1 were observed in cervical cancer patients with lower expression of *HOXA10* and *HOXA11*, indicative of the fact that *HOXA10* and *HOXA11* could be served as biomarkers of response to immunotherapy.

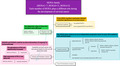

## 1 INTRODUCTION

Cancer has been the leading cause of death worldwide. Cervical cancer is the most common gynecological malignancy, the third most common malignant tumor in women worldwide, and the most common malignant tumor among Chinese women. According to a statistical report, a total of 65,620 patients were newly diagnosed with cervical cancer in 2020.^1^ The largest preventable cancer contributor in China is chronic infection, which is responsible for approximately 17% of all cancers in China^2^ and is predominantly comprised of human papillomavirus (HPV) for cervical cancer, *H*. *pylori* for stomach cancer, and HBV for liver cancer. The proven biological etiology of cervical cancer is HPV infection, in which HPV 16 and 18 are attributed to more than 70% of cases worldwide.^3^ The brush over of sexual education and economic backwardness of China are responsible for HPV infection. The most effective strategy for the prevention of infection‐related cancers is to create more effective vaccines against these carcinogenic viruses and to formulate better annihilation methods to combat these bacteria. In addition, appropriate screening methods are critical to improve the overall survival (OS) of cases. The adherence rates in the cervical cancer screening program were relatively high in the USA, with an adherence rate of 83%.^4^ At present, surgery, concurrent chemoradiotherapy and radiotherapy have better effects on early cervical cancer. Most of early stage of cervical cancer (stage I to IIa) can be cured by surgery or radiotherapy, while treatment should be tailored for advanced and metastatic cervical cancer, and concurrent chemoradiation is considered as the priority treatment. However, the efficacy is still limited.^5^ Therefore, the identification of biomarkers for early cervical cancer could be propitious to favor the outcome of cervical cancer.

Biomarkers play crucial roles in predicting the treatment response, prognosis, and disease progression in cancer, developing new therapies, and elucidating tumorigenesis mechanisms.^6^ The homeobox genes (*HOX*) are a series of genes coded crucial transcriptional regulators that play diverse roles from embryogenesis to tumorigenesis.^7^ In humans, the *HOX* family is arranged into four gene clusters termed *HOXA*, *HOXB*, *HOXC*, and *HOXD*.^7^ *HOXA* cluster genes include *HOXA1~7*, *HOXA9~11*, and *HOXA13*.^7^ Increasing evidence has shown that altered *HOXA* genes are involved in human cancer initiation and progression. *HOXA13* was expressed more in the normal colon tissues when compared with colon cancer, and *HOXA13* expression was associated with TNM stage of patients.^8^ *HOXA13* is also involved in HOTTIP‑induced malignant phenotypes of gastric cancer cells.^9^ It was also reported that *HOXA2* and *HOXA4* were downregulated and other *HOXA* members were upregulated in laryngeal squamous cell cancer, including *HOXA9* and *HOXA13*.^10^ In addition, upregulation of *HOXA10*, *HOXA11*, and *HOXA13* was correlated with poor laryngeal squamous cell cancer OS.^10^ However, to date, little is known about the expression profile and clinical significance of *HOXA* genes in cervical cancer.

In the present study, we analyzed different *HOXA* genes applying public database to determine their expression patterns, diagnostic, prognostic potential, and treatment values in cervical cancer.

## 2 MATERIALS AND METHODS

### 2.1 The cancer genome atlas (TCGA) data acquisition

The Cancer Genome Atlas (TCGA) projects (https://portal.gdc.cancer.gov/), consisting of excellent databases of very large RNA sequence data of cancerous and normal samples, provide great opportunities for high‐throughput modeling and bioinformatics analysis to determine diagnostic and prognostic biomarkers of cancer. The TCGA cervical cancer database includes a total of 306 cervical cancer and 3 normal control samples. We obtained high‐throughput sequencing (HTSeq) data, Illumina Human Methylation 450K data, and clinical parameters (including TNM stage, smoking history, grade, and age).

### 2.2 Expression and methylation data of *HOXA* genes in cervical cancer

The expression and methylation data of the *HOXA* genes were abstracted from HTSeq data and Illumina Human Methylation 450K data. The comparison of expression level of all *HOXA* genes was analyzed using an independent samples *t* test between cervical cancer and normal tissues.

### 2.3 Receiver operating characteristic (ROC) curve analysis

We performed a receiver operating curve (ROC) analysis to evaluate the diagnostic value of determination of *HOXA* expression in distinguishing cervical cancer patient and to calculate the area under the curve (AUC) value and cutoff value based on the maximum of Youden index.^11^ The Youden index is defined as sensitivity +specificity −1.^11^ Then, cervical cancer patients were grouped into two groups according to the cutoff of *HOXA* expression, respectively. Then, time‐dependent receiver operating characteristic (ROC) curve analyses and AUC were also performed to evaluate the prediction accuracy of our model.

### 2.4 Prognostic values of *HOXA* members in cervical cancer

The prognostic potential of the *HOXA* genes was assessed by Cox proportional hazards regression analyses. First, the associations between all clinical parameters (including *HOXA* genes expression, TNM stage, age, smoking history, and histological grade) and overall survival (OS) among cervical cancer patients were assessed using univariate Cox proportional hazards regression analyses. Subsequently, all the survival associated clinical features, including *HOXA* expression, were evaluated using multivariate Cox proportional hazards regression analysis.

### 2.5 Construction of nomograms

A nomogram generates a numerical probability of a clinical event based on a series of clinical features and a statistical predictive model.^12^ The development of a nomogram includes defining the patient outcomes, identifying important covariates, specifying the statistical model, and validating its performance. Briefly, the outcome is typically an event, which is the time to death in the current study. Nomograms were used to predict the probability of the 3‐ and 5‐year OS. The final model selection was performed using a backward step‐down process in the Cox proportional hazards regression analysis, that is, the prognostic covariates included the significant prognostic parameters assessed in our study.

Subsequently, a time‐dependent receiver operating characteristic (ROC) curve analysis involving the 3‐ and 5‐year OS was conducted to calculate the prognostic accuracy of the model for time‐dependent survival. The performance of the nomogram was measured using a concordance index (C‐index), which quantifies the level of concordance between the predicted and actual survival probabilities. The calibration was evaluated by plotting the relationship between actual and predicted probabilities using a bootstrapping method.^13^

### 2.6 Negative association between expression and methylation of the *HOXA* genes in cervical cancer

We also obtained the methylation levels of cg sites in the gene promoter regions of differentially expressed *HOXA* genes in cervical cancer. Then, we performed the Pearson's analysis to assess the association between *HOXA* expression and methylation in cervical cancer and applied the *corrplot* package to plot the association.

### 2.7 Immune cell abundance analysis

Immune Cell Abundance Identifier (ImmuCellAI) is an online tool to estimate the abundance of 24 immune cells from a gene expression dataset including RNA‐Seq and microarray data, in which the 24 immune cells comprise 18 T‐cell subtypes and 6 other immune cells: B cells, NK cells, monocytes, macrophages, neutrophils, and DCs.^14^ We used this tool to assess the infiltration difference in immune cells between the low‐ and high‐*HOXA* expression groups of cervical cancer. Besides, we also compared the expression of relevant immunity biomarkers (including programmed cell death protein 1 (PD‐1), programmed cell death ligand 1 (PD‐L1), and cytotoxic T lymphocyte‐associated antigen‐4 (CTLA4)) between low and high expression of *HOXA* groups.

### 2.8 Gene set enrichment analysis (GSEA)

The gene set enrichment analysis (GSEA) version 4.0.2 was developed by the Broad Institute. The c2.cp.v7.2.symbols.gmt (KEGG) was utilized and downloaded from the Molecular Signatures Database (MSigDB). Further comparisons of the enriched KEGG pathways for the high expression of *HOXA1* group and low expression of *HOXA10* and *HOXA11* groups were performed. Pathways with false discovery rate (FDR) <0.05 were considered with statistical significance.

### 2.9 Statistical analysis

The HTSeq data and methylation data of *HOXA* genes from the TCGA database were abstracted using Perl 5.28. The association between methylation and expression of *HOXA* members were assessed and plotted by the *corrplot* package, the *survival* package was used for the analysis of prognostic values, and the *rms* package was applied for the construction of the nomogram. The comparison of expression of *HOXA* genes in cervical cancer tissues and normal controls was analyzed using independent samples *t* test by SPSS 25.0.

## 3 RESULTS

### 3.1 Expression profile of *HOXA* family in cervical cancer

**FIGURE 1:**
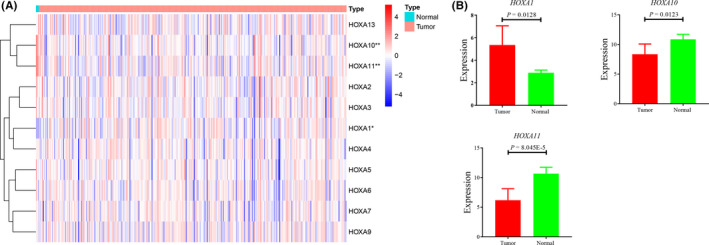
Heatmap analysis of *HOXA* members expression in cervical cancer (A) and comparison of *HOXA1*, *HOXA10*, and *HOXA11* expression with significant statistically in cervical cancer (B). N _(tumor)_ = 306; N _(normal)_ = 3

To obtain a full picture of the expression status of *HOXA* members in cervical cancer, we downloaded the HTSeq data of *HOXA* expression, which originated from the TCGA database (https://portal.gdc.cancer.gov/). The expression profile of *HOXA* family was presented by *pheatmap* of R software (Figure 1A), the result showing significant differences in *HOXA1*, *HOXA10*, and *HOXA11* expression between cervical cancer and normal controls. Subsequently, we performed independent samples *t* test to specific the difference of expression. Our results demonstrated that the expression of *HOXA1* was upregulated in cervical cancer (Figure 1B, *p* value for *HOXA1* = 0.0128), while *HOXA10* and *HOXA11* expression was downregulated in cervical cancer compared with the normal control (Figure 1C,D; *p* value for *HOXA10* = 0.0123, *p* value for *HOXA11* = 8.045E‐5). The rest of the *HOXA* members (including *HOXA2*‐*9* and *HOXA13*) showed very small differences between cervical cancer and normal controls, with no statistical significance (Figure S1).

### 3.2 Diagnostic value of *HOXA* members in cervical cancer

**FIGURE 2:**
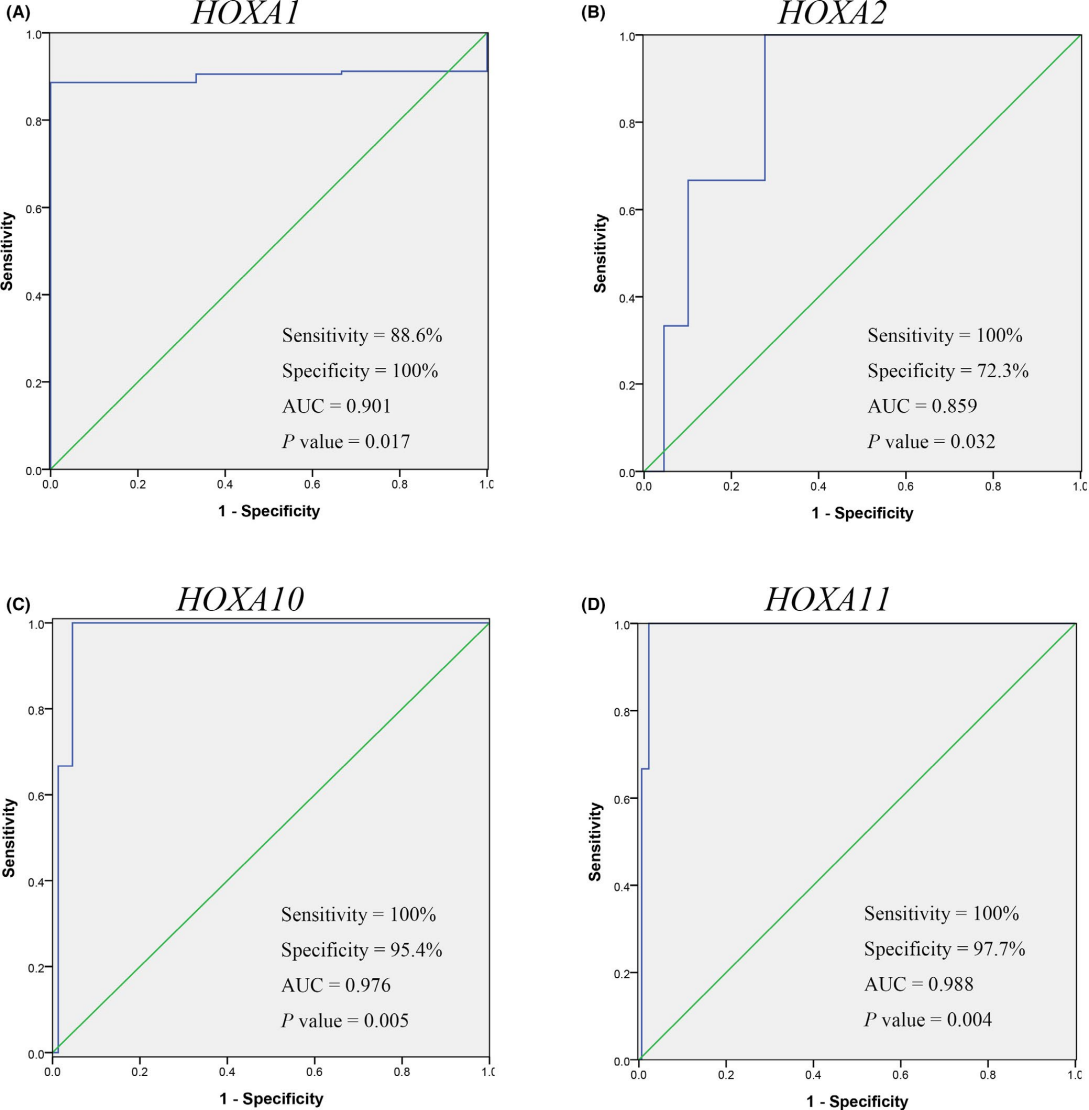
Receiver operating characteristic (ROC) curve with area under the curve (AUC) plots for *HOXA1*, *HOXA2*, *HOXA10*, and *HOXA11* in cervical cancer

To evaluate the discriminative ability of *HOXA* members, a receiver operating characteristic (ROC) curve with an area under the curve (AUC) was built. The results showed that *HOXA1*, *HOXA2*, *HOXA10*, and *HOXA11* expression achieved specificity rates of 100%, 72.3%, 95.4%, and 97.7% with sensitivity rates greater than 95%, and the AUCs were 0.901, 0.859, 0.976, and 0.988, respectively (Figure 2). The rest of *HOXA* members showed slight diagnostic value in distinguishing of cervical cancer (Figure S2).

### 3.3 Prognostic value of *HOXA* members in cervical cancer

**FIGURE 3:**
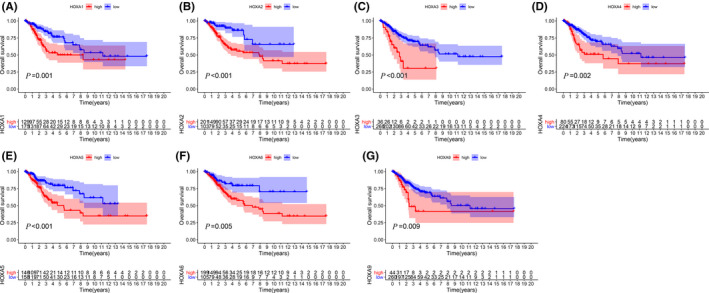
Kaplan‐Meier plots of *HOXA* members (*HOXA1*, *HOXA2*, *HOAX3*, *HOXA4*, *HOXA5*, *HOXA6*, and *HOXA9*) in cervical cancer

Furthermore, the prognostic values of *HOXA* members were estimated. Firstly, the *survival* package of R software was utilized to execute the Kaplan‐Meier plot. The results were exhibited in Figure 3 and revealed that low expression of seven *HOXA* members were correlated with favor overall survival of cervical cancer (including *HOXA1*, *HOXA2*, *HOAX3*, *HOXA4*, *HOXA5*, *HOXA6*, and *HOXA9*). Considering the principle of the *survival* package (seeking the optimal cutoff for overall survival, grouping patient in two group, and then drawing the Kaplan‐Meier plot), we then determined the predictive potential of *HOXA* expression using continuous expression by Cox proportional hazards regression analyses.

First, we performed univariate Cox proportional hazards regression analysis, and the results showed that TNM stage and the expression of *HOXA1*, *HOXA2*, *HOXA3*, and *HOXA4* were associated with poor outcome in cervical cancer patients (hazard ratio [HR] for T3 vs T1: 2.668 (1.149–6.194), HR for T4 vs T1: 8.093 (3.425–19.127), HR for N+ vs N−: 2.897 (1.473–5.696), HR for M+ vs M−: 3.641 (1.223–10.844), HR for *HOXA1*: 1.908 (1.234–2.948), HR for *HOXA2*: 1.659 (1.004–2.741), HR for *HOXA3*: 1.677 (1.112–2.526), and HR for *HOXA4*: 1.681 (1.023–2.763), Table 1). Subsequently, to avoid noise in OS caused by the TNM stage of cases, we applied multivariate Cox proportional hazards regression analysis. After normalization of the other clinical features (including TNM stage), only *HOXA1* and *HOXA3* expression was still associated with poor OS of cervical cancer, indicative of the fact that these two *HOXA* members could serve as independent predictive biomarkers of cervical cancer (Tables 2 and 3).

**TABLE 1:** Univariate analysis of *HOXA* gene expression and clinical features in cervical cancer

Parameter	Univariate analysis		
	Hazard ratio	95% CI	*p*
Age	1.011	0.990–1.032	0.307
Smoking history			
Negative (ref)	–	–	–
Positive	0.788	0.312–1.992	0.615
M stage			
M− (ref)	–	–	–
M+	3.641	1.223–10.844	0.021
N stage			
N− (ref)	–	–	–
N+	2.897	1.473–5.696	0.002
T stage			
T1 (ref)	–	–	–
T2	1.139	0.556–2.335	0.720
T3	2.668	1.149–6.194	0.022
T4	8.093	3.425–19.127	2.00E‐06
Stage			
Stage I (ref)	–	–	–
Stage II	1.017	0.493–2.097	0.963
Stage III	0.803	0.282–2.283	0.681
Stage IV	5.438	2.739–10.796	1.00E‐06
Grade			
G1 (ref)	–	–	–
G2	1.289	0.395–4.201	0.673
G3	1.092	0.326–3.661	0.885
*HOXA1* expression	1.908	1.234–2.948	0.0036
*HOXA2* expression	1.659	1.004–2.741	0.048
*HOXA3* expression	1.677	1.112–2.526	0.013
*HOXA4* expression	1.681	1.022–2.763	0.040
*HOXA5* expression	1.295	0.986–1.700	0.062
*HOXA6* expression	1.402	0.987–1.990	0.058
*HOXA7* expression	1.327	0.856–2.057	0.204
*HOXA9* expression	1.232	0.989–1.533	0.061
*HOXA10* expression	1.111	0.814–1.517	0.504
*HOXA11* expression	1.069	0.809–1.411	0.639
*HOXA13* expression	1.014	0.771–1.333	0.921

**TABLE 2:** Multivariate analysis of *HOXA1* expression in cervical cancer

Parameter	Multivariate analysis		
	Hazard ratio	95% CI	*p*
T stage			
T1 (ref)	–	–	–
T2	0.844	0.390–1.827	0.668
T3	2.308	0.894–5.956	0.083
T4	5.286	1.733–16.125	3.43E−03
N stage			
N− (ref)	–	–	–
N+	2.765	1.389–5.504	0.0037
M stage			
M− (ref)	–	–	–
M+	2.119	0.617–7.276	0.232
*HOXA1* expression	1.870	1.175–2.976	0.0082

**TABLE 3:** Multivariate analysis of *HOXA3* expression in cervical cancer

Parameter	Multivariate analysis		
	Hazard ratio	95% CI	*p*
T stage			
T1 (ref)	–	–	–
T2	0.825	0.383–1.779	0.625
T3	2.089	0.818–5.333	0.123
T4	6.697	2.200–20.390	8.14E−04
N stage			
N− (ref)	–	–	–
N+	3.016	1.507–6.034	0.0018
M stage			
M− (ref)	–	–	–
M+	1.765	0.531–5.871	0.353
*HOXA3* expression	1.666	1.131–2.453	0.0096

### 3.4 Prognostic nomogram for OS in cervical cancer

**FIGURE 4:**
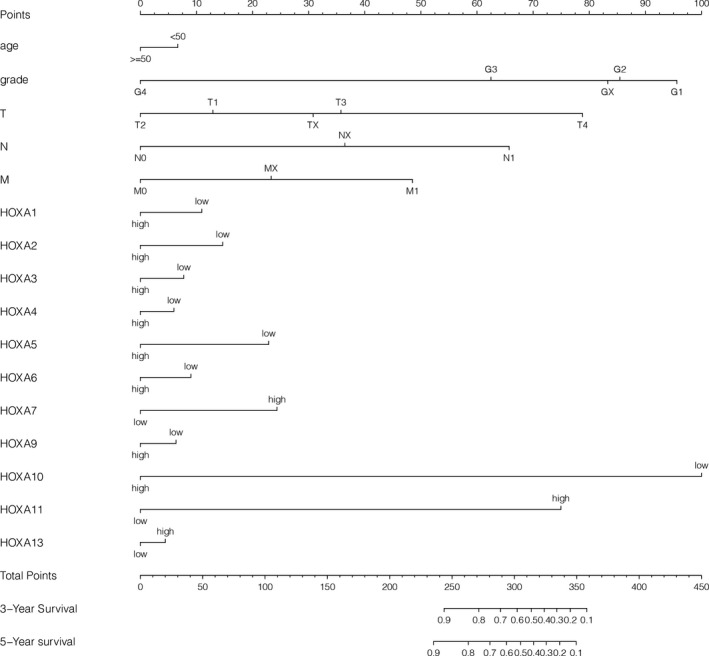
Nomogram for the prediction of 3‐ and 5‐year OS among cervical cancer patients

**FIGURE 5:**
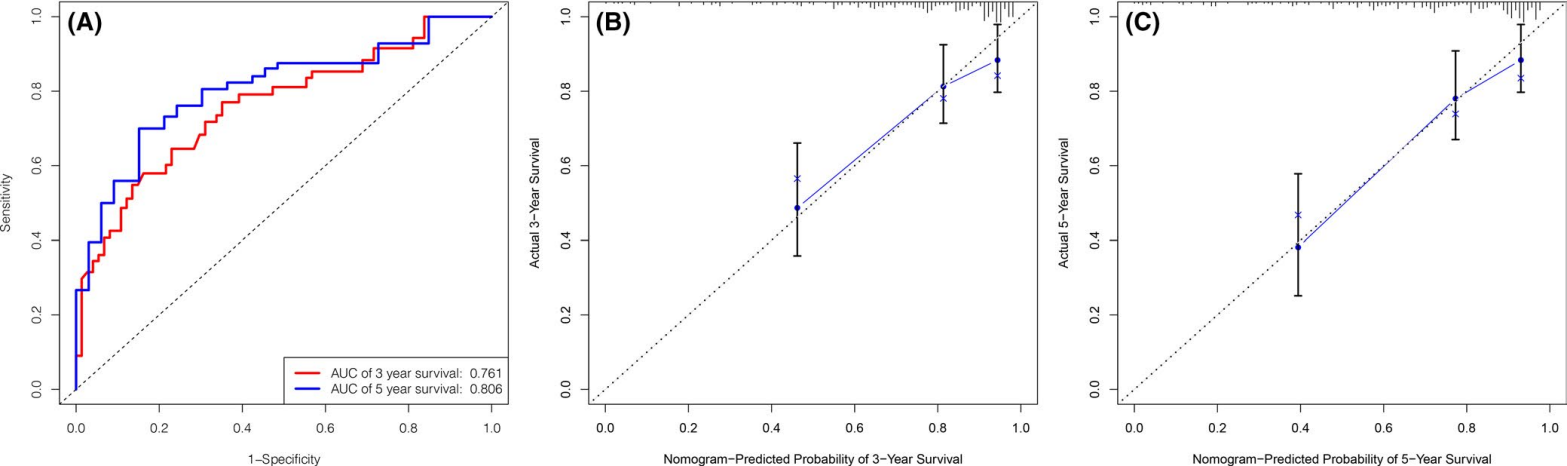
Calibration curves for predicting the OS of cervical cancer patients at 3 and 5 years in the original cervical cancer cohort (A) and at (B) 3 and (C) 5 years in the validation cohort

Predictive models with nomograms were constructed using the *rms* package in R software, integrating age, histological grade, TNM stage, and *HOXA* expression, as shown in Figure 4. Each prognostic parameter was assigned a score according to its prognostic value; the sum total of the scores was used to predict 3‐ and 5‐year OS. The total score for all the variables was converted into an estimate of the probability of death. The performance of the nomogram was measured by the concordance index (C‐index); the larger the C‐index, the more accurate the prognosis. The C‐index for overall survival prediction was 0.763. The AUCs for the 3‐ and 5‐year OS were 0.761 and 0.806, respectively (Figure 5A). The AUC combined with the C‐index reflected the good discrimination ability of the model. The calibration plots showed good agreement between the actual and predicted probabilities of both the 3‐ and 5‐year OS (Figure 5B,C).

### 3.5 Negative correlation of *HOXA* expression and promoter methylation

**FIGURE 6:**
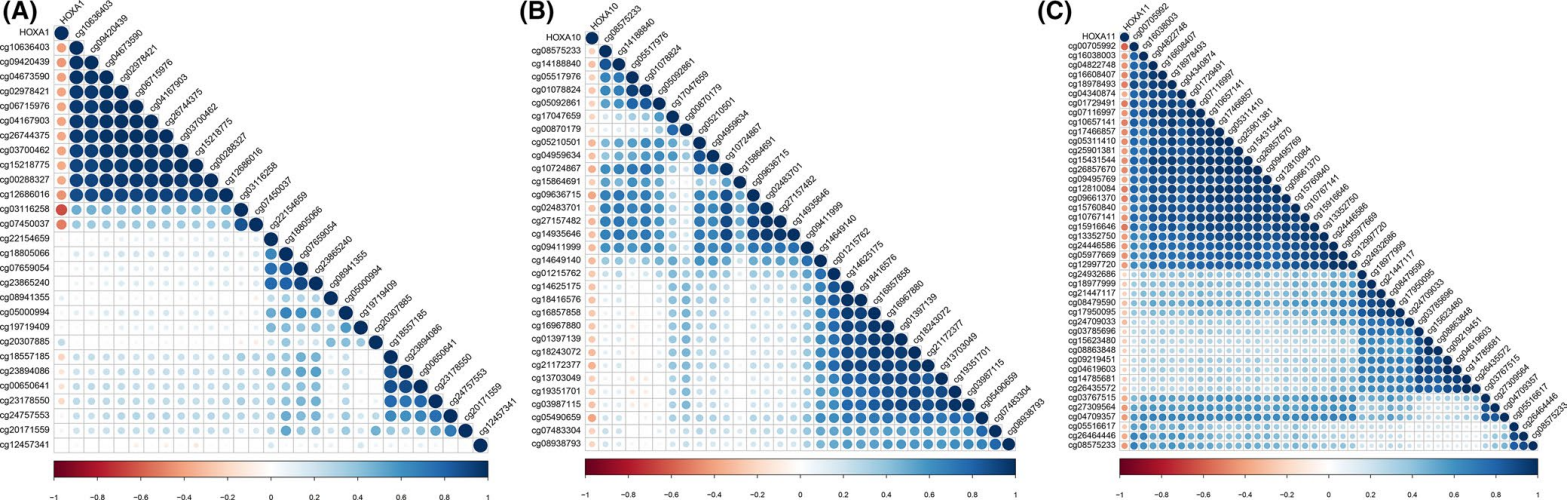
Negative correlation of *HOXA1* (A), *HOXA10* (B), and *HOXA11* (C) methylation and expression

To create multiple views of the roles of the *HOXA* family in cervical cancer, we simultaneously obtained the methylation data of *HOXA* members. We identified three differentially expressed *HOXA* members in cervical cancer (upregulated *HOXA1* and downregulated *HOXA10* and *HOXA11*). Pearson's correlation results showed that almost all assessed cg sites of *HOXA1, HOXA10*, and *HOXA11* exhibited a negative correlation with expression in cervical cancer (Figure 6A–C).

### 3.6 Immune cell abundance analysis

**FIGURE 7:**
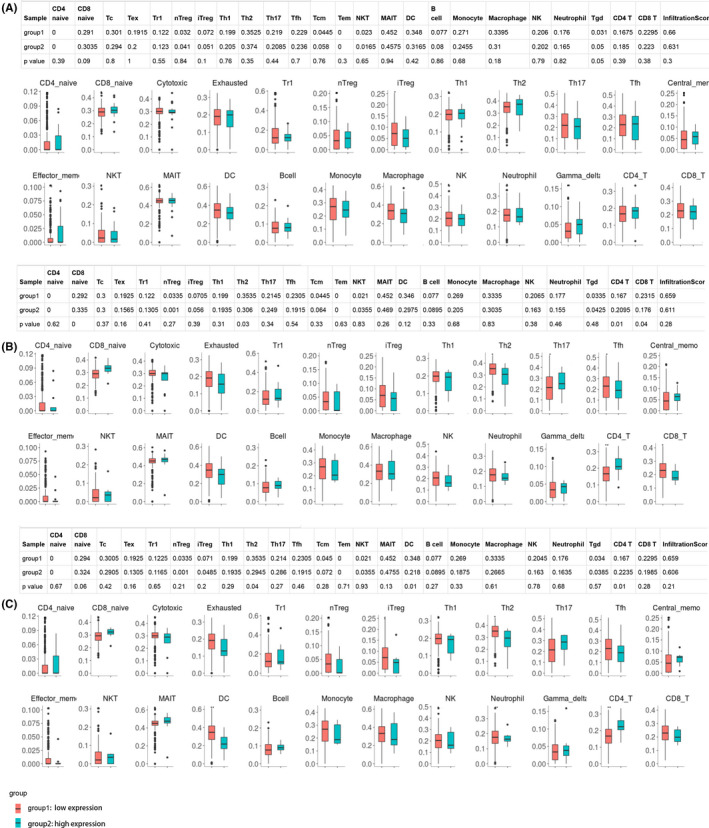
Immune cell abundance analysis between two groups based on different expression levels of *HOXA1* (A), *HOXA10* (B), and *HOXA11* (C)

**FIGURE 8:**
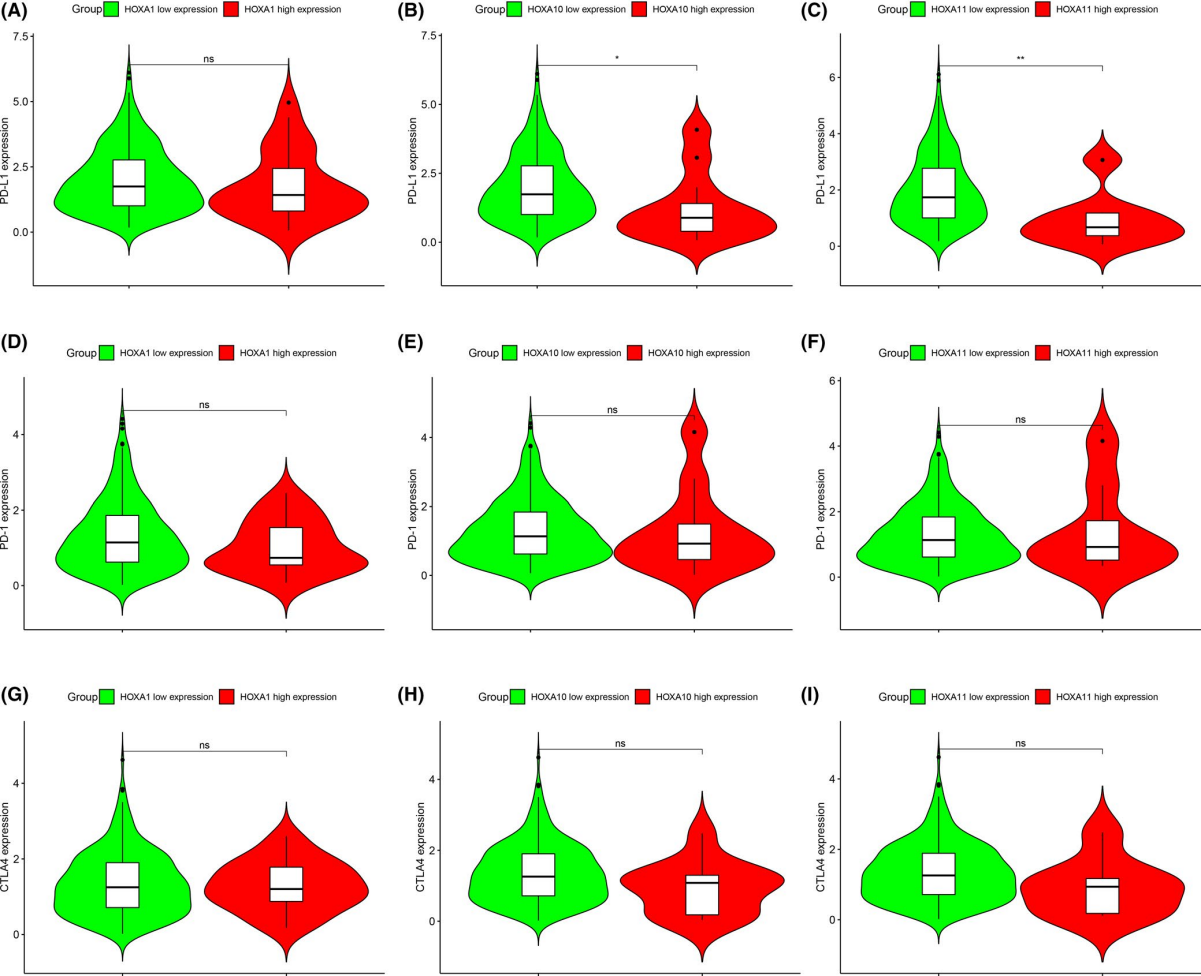
Differential expression of immune checkpoint inhibitor (ICI) biomarkers between two groups based on different expression levels of *HOXA1*, *HOXA10*, and *HOXA11*

Firstly, patients with cervical cancer were classified into two different group based on the cutoff value of *HOXA1*, *HOXA10*, and *HOXA11* expression from ROC. We analyzed the infiltration difference in immune cells between the low‐ and high‐*HOXA1*, *HOXA10*, and *HOXA11* expression groups using the ImmuCellAI online tool. We found that the abundance of T helper 2 cells (Th2) was significantly increased in both the low‐*HOXA10* and *HOXA11* expression groups, while the abundance of dendritic cells (DCs) was increased in the low‐*HOXA11* expression group (Figure 7B,C). There was no difference in the infiltration of immune cells between the low‐ and high‐*HOXA1* expression groups (Figure 7A). Subsequently, we also analyzed the difference expression of several immunity biomarkers (CTLA4, PD‐1, and PD‐L1) between the low‐ and high‐*HOXA1*, *HOXA10*, and *HOXA11* expression groups. Our results showed that only patients with low expression of *HOXA10* and *HOXA11* were characterized with relatively high expression of PD‐L1 (Figure 8). This phenomenon conveys a very important message that the application of immune checkpoint inhibitor (such as anti‐PD‐L1) could be served as effective treatment to ameliorate the overall survival of cervical cancer.

### 3.7 Analyses of the signaling pathways participated in cervical cancer using GSEA

**FIGURE 9:**
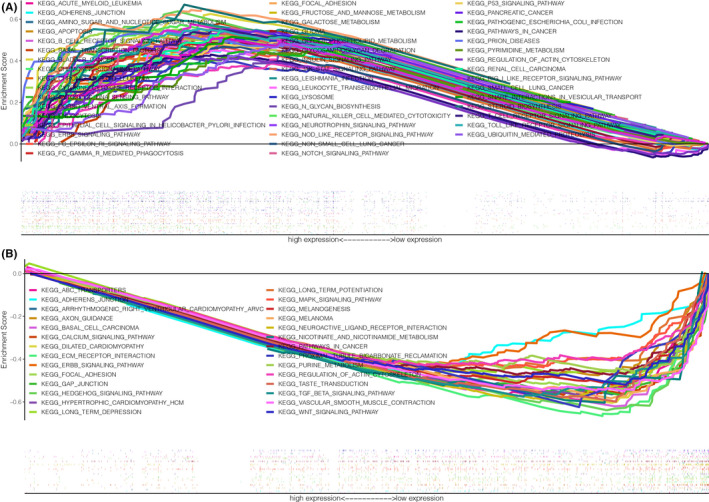
The Gene Set Enrichment Analysis (GSEA) of the relationship between the expression level of *HOXA1* and *HOXA11* in cervical cancer

The GSEA results revealed that multiple cancer‐related pathways (including NOTCH signaling, p53 signaling, apoptosis pathway, and pathways in cancer) were enriched in the cervical cancer patients with high expression of *HOXA1* and low expression of *HOXA11* (Figure 9, detail information shown in Tables S1 and S3). Among these associated pathways, the immune‐related pathways were upregulated (such as B‐cell receptor signaling pathways, T‐cell receptor signaling, Toll‐like receptor signaling pathway) in cervical cancer cases with high expression of *HOXA1*, whereas little of enriched KEGG pathways was observed in cervical cancer cases with high expression of *HOXA10* (Table S2).

## 4 DISCUSSION

Homeobox (HOX) genes were firstly observed in the fruit fly, a large family of genes characterized by the presence of a conserved DNA sequence.^7^ HOX genes encode a family of evolutionarily conserved homeodomain transcription factors that are crucial during both development and adult life.^15^ In humans, a total of 39 *HOX* genes are arranged in four clusters, namely *HOXA*, *HOXB*, *HOXC*, and *HOXD*, which are located on various chromosomes.^16^ Each cluster is divided into 13 paralog groups (*HOXA1‐13* genes) based on the position of chromosomes.^17^ These genes are characterized by a consensus DNA sequence.^18^ Evidence of multiple studies has identified biological functions for *HOXA* genes during morphogenesis, patterning, and differentiation.^19^, ^20^, ^21^ It was reported that *HOXA* is the most highly expressed in the developing lungs of mice^22^, ^23^; however, mice with mutations in *HOXA5* at birth lack pulmonary surfactant, indicating a role of *HOXA5* gene in morphogenesis.^24^

Abnormal expression of *HOXA* genes has been proven in multiple cancers.^25^, ^26^ The oncogenic potential of *HOXA* genes has been clearly implicated in leukemia, and their roles in other neoplasia are also being evaluated.^27^ The increased expression of *HOXA9* could be found in most aggressive leukemia,^28^ and *HOXA13* physically linked to tumor growth and angiogenesis.^29^ Numerous reports have cataloged differences in *HOXA* gene expression between normal and neoplastic tissues. *HOXA1* and *HOXA7* were expressed in small cell lung cancer, while they were not expressed in normal lungs. Therefore, the authors proposed that the *HOXA* gene profiles of cells obtained from sputum could act as molecular markers to aid the detection of lung carcinoma.^30^ Upregulation of *HOXA10* expression plays a key role in colorectal cancer development and could be considered as a new biomarker that indicates poor prognosis.^31^ These studies supported the monitor potential of *HOXA* genes in the diagnosis and prognosis of cancers.

However, the expression profile of the *HOXA* genes in cervical cancer was not comprehensively represented. This study showed the expression profile of *HOXA* genes in cervical cancer, and our results represented the expression difference of three members of *HOXA* genes (*HOXA1*, *HOXA10*, and *HOXA11*) between the cervical cancer group and the normal control group with statistically significance (*p* value for *HOXA1* = 0.0128; *p* value for *HOXA10* = 0.0123, *p* value for *HOXA11* = 8.045E‐5). The expression of *HOXA1* was significantly upregulated in cervical cancer compared to normal control, whereas *HOXA10* and *HOXA11* were downregulated in cervical cancer. In addition, our results showed that the expression level of *HOXA* members (*HOXA1*, *HOXA10*, and *HOXA11*) are affected by the methylation level, which has been reported by previous study.^10^, ^32^

A previous study identified *HOXA4* as the only prognostic gene in acute myeloid leukemia (AML), the upregulation of which in patients with normal karyotypes was actually associated with longer‐term survival.^28^ *HOXA13* may serve as a prognostic parameter in kidney renal clear cell carcinoma patients.^33^ In our research, we also assessed the prognostic ability of the *HOXA* family in cervical cancer patients. A notable phenomenon was presented, which awaits the emergence of further large‐scale sample studies involving the *HOXA* gene family. Our results showed that among three differentially expressed *HOXA* genes (*HOXA1*, *HOXA10*, and *HOXA11*), *HOXA1* was the only prognostic gene in cervical cancer. However, among the remaining members of the *HOXA* family, *HOXA3* also exhibited prognostic capability without differential expression in cervical cancer. We also developed a clinical tool (a nomogram) that predicts the survival of cervical cancer. A nomogram is a simple graphical representation of a statistical predictive model that estimates the individualized risk of a clinical event (3‐ and 5‐year OS in the current study); nomograms have recently emerged as accurate tools for estimating prognosis in oncology.^34^, ^35^ Our nomogram is simple to use and would be useful for estimating the OS of patients with cervical cancer. It visualizes the associations among each *HOXA* member, the TNM stage, age, histological grade, and the tumor prognosis of cervical cancer patients. However, in the near future, clinical and pathological features could be integrated with genomic data to further improve the predictive ability of the model.

Increasing evidence suggests that immune cells play critical roles in carcinogenesis and progression, and a proper proportion of T‐cell subsets could contribute to the long‐term clinical benefits of anticancer treatments.^36^, ^37^ In this study, we applied the online tool ImmuCellAI, a highly accurate method of estimating the abundance of immune cells.^14^ Our results showed that under the significantly decreased abundance of CD4+ T cells, the abundance of Th2 cells was still remarkably increased in both the low‐*HOXA10* and *HOXA11* expression groups. T cells can be divided into two major subgroups, CD4+ and CD8+, according to the expression of cell surface differentiation antigens. Th2 belongs to the subset of T helper CD4+ T cell. CD4+ T‐cell suppression or dysfunction has been reported as the mechanism causing cancer escape; the Th2 response was associated with tumor immune evasion in mouse studies,^38^, ^39^ known to be particularly powerful in promoting tumor progression and suppressing antitumor immune responses. In our study, decreased expression of *HOXA10* and *HOXA11* was found in cervical cancer samples when compared with their expression in normal control samples, and the infiltration of immune cells analyzed found the imbalance of Th1 and Th2, implying that the dysfunction of *HOXA* family might affect the cancer escape of immunity. Considering favorable efficacy in immune checkpoint inhibitors (especially PD‐1/PD‐L1 inhibitors) achieved in treating cervical cancer,^40^ our study also assessed the expression status of CTLA4, PD‐1, and PD‐L1 in cervical cancer patients grouped by different expression of *HOXA1*, *HOXA10*, and *HOXA11*. The cell surface receptor PD‐1 is expressed by T cells on activation during priming or expansion and binds to one of two ligands, PD‐L1 and PD‐L2.^41^, ^42^ Many types of cells can express PD‐L1, including tumor cells; binding of PD‐L1 to PD‐1 generates an inhibitory signal that attenuates the activity of T cells. The PD‐L1/PD‐1 axis was found to be an important negative feedback loop that ensures immune homeostasis; it is also an important axis for restricting tumor immunity.^42^ In our study, higher expression of PD‐L1 was observed in cervical cancer patients with low‐*HOXA10* and *HOXA11* expression. These results provided evidence that *HOXA10* and *HOXA11* could be served as effective molecular biomarkers for prognosis of sensitivity of immunotherapy. Besides, the results of GSEA identified multiple immune‐related pathways enriched in cervical cancer patients.

In summary, this study represented the expression status of *HOXA* members in cervical cancer and identified three differentially expressed *HOXA* genes (*HOXA1*, *HOXA10*, and *HOXA11*) with great discriminative ability in cervical cancer. The *HOXA1* gene could serve as an independent prognostic factor for poor OS in patients with cervical cancer. In addition, preliminarily analysis showed that the expression of *HOXA10* and *HOXA11* could be served as biomarkers of response to immunotherapy, which needs further verification by randomized controlled trials with large sample sizes. Our findings may inspire new clinical practices for patients with cervical cancer, including diagnosis, treatment, and prognosis.

## CONFLICT OF INTEREST

The authors declare that there are no conflicts of interest.

## AUTHOR CONTRIBUTIONS

FG and JZ were responsible for designing the research study. YZ, WT, and YF were in charge of analyzing the data from public database. FG was responsible for writing of article. All authors reviewed the study.

## Supporting information

Fig S1

Fig S2

Tab S1

Tab S2

Tab S3

## Data Availability

The data that support the findings of this study are openly available in TCGA database at https://portal.gdc.cancer.gov/.
