# Supplementary figures and images for: Expression of the HOXA gene family and its relationship to prognosis and immune infiltrates in cervical cancer
Source: J Clin Lab Anal. 2021 Oct 4;35(11):e24015. doi: 10.1002/jcla.24015 (PMC8605136; doi:10.1002/jcla.24015)

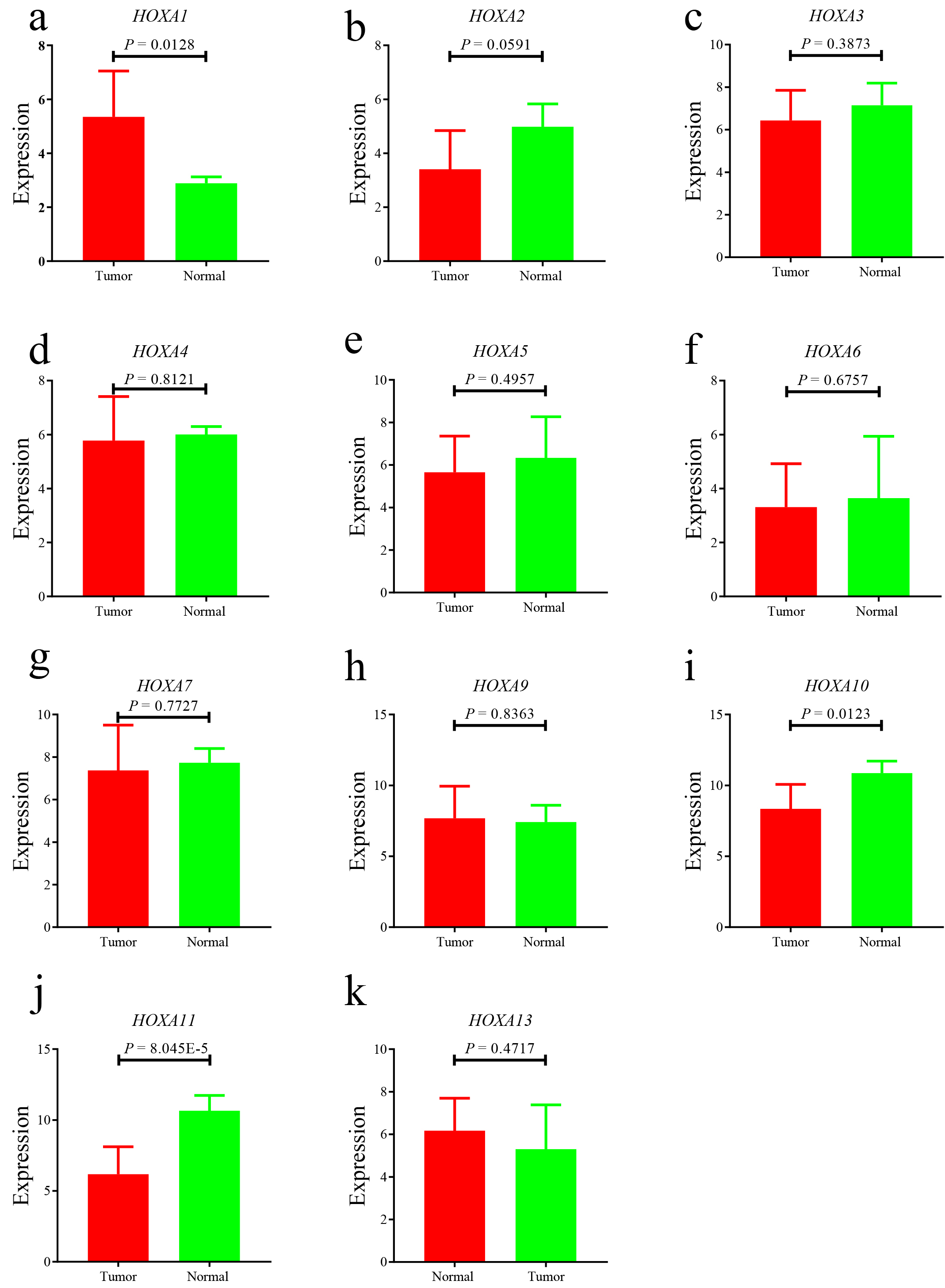

Supplement: Supplementary file 1 — Fig S1 [file JCLA-35-e24015-s005.jpg]

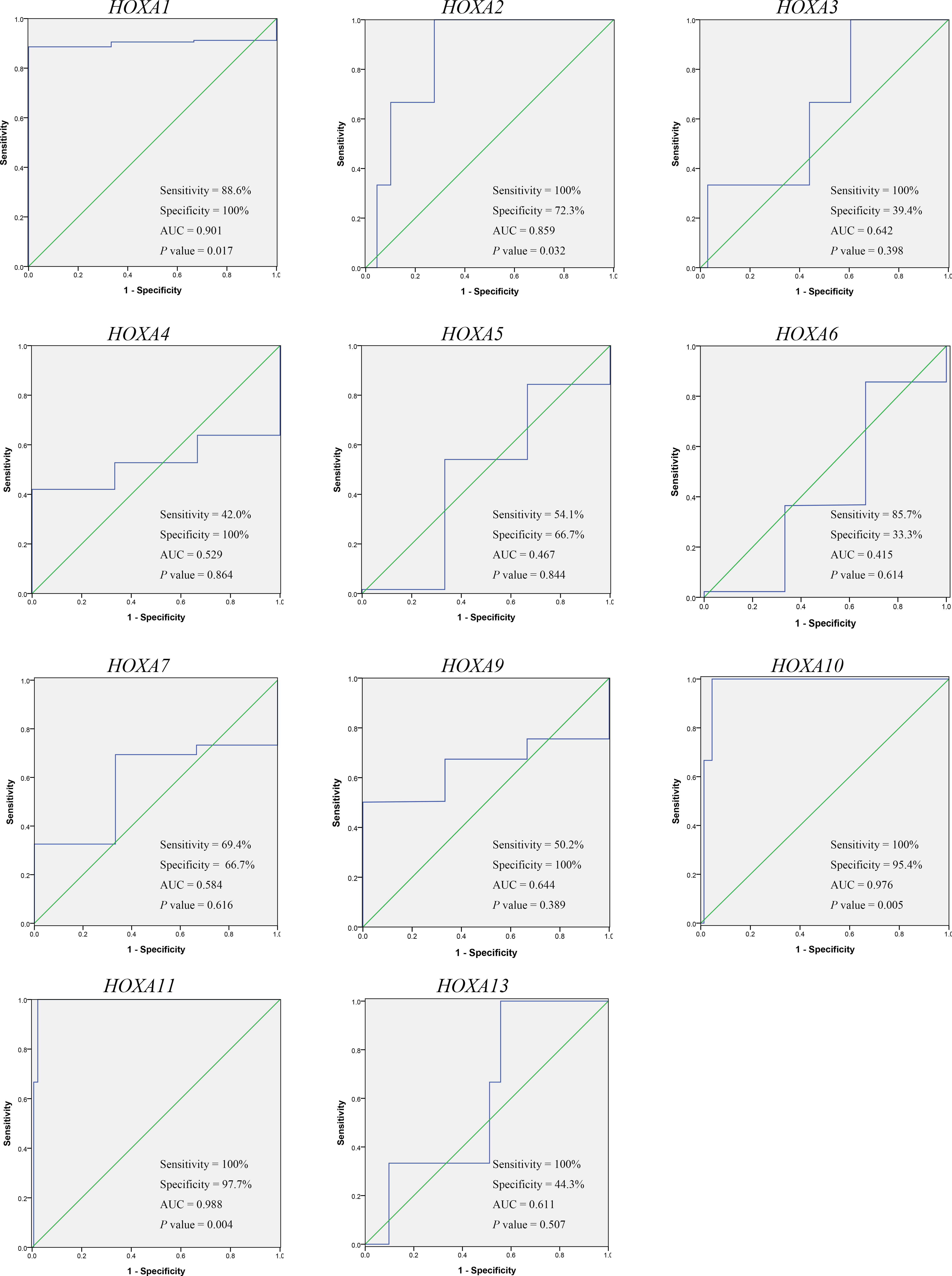

Supplement: Supplementary file 2 — Fig S2 [file JCLA-35-e24015-s003.jpg]
